# Heavy Metal Contamination of Vegetables Irrigated by Urban Stormwater: A Matter of Time?

**DOI:** 10.1371/journal.pone.0112441

**Published:** 2014-11-26

**Authors:** Minna Tom, Tim D. Fletcher, David T. McCarthy

**Affiliations:** 1 Environmental and Public Health Microbiology Lab (EPHM Lab), Department of Civil Engineering, Monash University, Melbourne, Victoria, Australia; 2 Waterway Ecosystem Research Group, School of Ecosystem and Forest Sciences, Faculty of Science, University of Melbourne, Melbourne, Victoria, Australia; Estación Experimental del Zaidín (CSIC), Spain

## Abstract

Urban stormwater is a crucial resource at a time when climate change and population growth threaten freshwater supplies; but there are health risks from contaminants, such as toxic metals. It is vitally important to understand how to use this resource safely and responsibly. Our study investigated the extent of metal contamination in vegetable crops irrigated with stormwater under short- and long-term conditions. We created artificially aged gardens by adding metal-contaminated sediment to soil, simulating accumulation of metals in the soil from irrigation with raw stormwater over zero, five and ten years. Our crops - French bean (*Phaseolus vulgaris*), kale (*Brassica oleracea* var. *acephala*), and beetroot (*Beta vulgaris*) - were irrigated twice a week for 11 weeks, with either synthetic stormwater or potable water. They were then tested for concentrations of Cd, Cr, Pb, Cu and Zn. An accumulation of Pb was the most marked sign of contamination, with six of nine French bean and seven of nine beetroot leaf samples breaching Australia's existing guidelines. Metal concentration in a crop tended to increase with the effective age of the garden; but importantly, its rate of increase did not match the rate of increase in the soil. Our study also highlighted differences in sensitivity between different crop types. French bean demonstrated the highest levels of uptake, while kale displayed restrictive behaviour. Our study makes it clear: irrigation with stormwater is indeed feasible, as long as appropriate crops are selected and media are frequently turned over. We have also shown that an understanding of such risks yields meaningful information on appropriate safeguards. A holistic approach is needed - to account for all routes to toxic metal exposure, including especially Pb. A major outcome of our study is critical information for minimising health risks from stormwater irrigation of crops.

## Introduction

Urban agriculture is increasingly adopted around the world, for the production of food crops, and given its many benefits to the community and the environment it is probably here to stay [1]. But availability of water has been a serious limiting factor [1], prompting a search for alternative resources. Wastewater, grey water and stormwater offer solutions, and reduce the significant pressure of population growth and climate change on our previous freshwater resources [2].

Stormwater is an especially abundant resource, which is currently only harvested for low-risk end-uses such as toilet flushing or garden irrigation [3]. While its harvest for higher-risk end-uses is uncommon, it has great potential, especially for economic and sustainable production of food crops, as opposed to traditional scarce and expensive sources. Indeed, it is generated close to the end-use, so apart from merely reducing its presence as a hazard to the built environment, there are many positive benefits from harvesting it [4], [5].

Direct use of stormwater is constrained, however, by typically high and variable pollution levels [6]–[8]. The toxicity and concentration profile of heavy metals in stormwater is of particular concern [9]. Although many metals are essential micronutrients for humans, they are toxic in excess [10], [11]. Other metals such as As, Cd, Hg and Pb are not essential, and are known to be harmful if exposure exceeds certain limits [12], [13]. Adverse health impacts from chronic ingestion of these metals may only become apparent after several years [14], [15].

Previous research has demonstrated that crops irrigated with contaminated water sources may be unsafe for human consumption [16], [17]. Metal uptake in crops is regulated by several factors, including bioavailability of the metal in soil, crop type [18]–[22] and metal distribution in the crop [23].

Long-term application of contaminated irrigation waters can also result in the accumulation of metals in soil [18], [22], [24]. The speciation of metals (the chemical form in which they are present) affects their bioavailability, and with longer residence in soils they undergo time-dependent chemical processes that may render them unavailable for uptake by plants [25]–[28]. Because they create an unrealistically rich pool of bioavailable metals, attempts to represent and understand metal ecotoxicity through the dosing of soils with laboratory-grade metals are of limited practical value [29]–[30]. Processes in a plant's metal uptake may be similar with water from diverse sources; but stormwater has unique and inherently variable characteristics, and these need careful attention.

Only one study has specifically investigated risks in stormwater irrigation of food crops [31]. They collected stormwater from an urban carpark, treated this using a gravity-fed vegetated sand filter [32] and stored this treated water in open and closed water tanks prior to being used to irrigate edible crops via spray and drip irrigation. They found elevated concentrations of both lead and cadmium in their edible vegetables, yet the source of this contamination was unclear (contaminated soil, atmosphere, or the stormwater). Further research is required to understand these sources, and the impact of using urban stormwater results to irrigate edible crops.

This study investigates the metal contamination of edible crops irrigated with urban stormwater. Short- and long-term risks are assessed in a semi-controlled laboratory environment. To naturalise the simulation of long-term stormwater irrigation, a new compressed timescale method is used to reproduce the metal accumulation that may occur from long-term stormwater irrigation. Crops irrigated with potable water for equivalent periods are used as a control. The study succeeds in providing valuable information about potential health risks associated with using stormwater for crop irrigation.

## Materials and Methods

### Study site and experimental set-up

The outdoor laboratory study was undertaken in an urban environment in Clayton, Melbourne, Australia. Four metal grid units coated with rustproof paint were used, each grid containing twenty-five 30 cm ×30 cm planting cells, 60 cm deep. Each cell was fitted with a planter bag (high density plastic) that extended to fill the space. A small outflow pipe at the bottom of each cell enabled drainage. The planting cells represented gardens, where all gardens allocated to kale and French bean were planted with a single crop. For beetroot, five crops were sown per garden and spaced 15 cm apart.

From bottom to top, the media profile of each cell was: a 170 mm layer of 7 mm scoria, a geotextile sheet, 40 mm layer of coarse river sand, 45 mm layer fine white wash sand and a top 300 mm layer of composted manure soil. Aside from the top layer, this profile and these depths were based on proportions recommended for use in a stormwater raingarden [33]. The composted manure soil was selected for its high organic and nutrient content [34]. This design tested the impact of three main factors on the uptake of heavy metals in crops (see Table 1): type of water source (potable water versus synthetic stormwater; 2 values), effective “age” of the garden (representing the level of contamination already in the soil; 3 values), and type of crop (3 values). The age groups were used to reflect the accumulated metal mass - from zero, five and ten years of irrigation for each water source. The crops used were kale (*Brassica oleracea* var. *acephala*), beetroot (*Beta vulgaris*) and French bean (*Phaseolus vulgaris*), representing the leafy, root and legume vegetable groups respectively. The entire experiment therefore consisted of 18 treatments (2×3×3) each with 3 replicates, making a total of 54 planting cells.

**Table 1:** Treatment matrix for the experiment.

Water source type	Age (yrs)	Crop type
**Potable water**	0	Kale
	0	Beetroot
	0	French bean
**Synthetic stormwater**	0	Kale
	0	Beetroot
	0	French bean
**Potable water**	5	Kale
	5	Beetroot
	5	French bean
**Synthetic stormwater**	5	Kale
	5	Beetroot
	5	French bean
**Potable water**	10	Kale
	10	Beetroot
	10	French bean
**Synthetic stormwater**	10	Kale
	10	Beetroot
	10	French bean

To avoid cross-contamination, two grids comprised only potable water cells and two grids comprised only stormwater cells. Beyond that constraint, placement of each replicate was random in the grids - to minimise environmental and dosing biases. The set-up was covered in nylon netting so the crops would not be damaged by birds or possums.

A novel “ageing” method was employed to establish treatments that represented metal accumulation in soils from long-term irrigation with potable water and stormwater. Three levels were established: zero years (new/unaged soil), five years and ten years of irrigation. Each ageing level represented an increase in the soil metal concentrations proportional to the increase in irrigation time (i.e. zero, five or 10 years) with the respective water source. For example, the soil metal concentrations in the stormwater ten years treatment would be approximately twice the soil metal concentrations in the stormwater five years treatment.

An estimate of the mass of heavy metals in the soils of the five- and ten-year “aged” cells was based on a weekly irrigation volume of 2.6 L for each cell (area  = 0.09 m^2^), using Food and Agricultural Organisation (FAO) guidelines on crop water requirements [35]. Typical heavy metal concentrations for urban stormwater have been established in the literature [7], [36]–[55], and also for the local potable water [56]. Together with irrigation volumes, these data set the target metal concentrations to be achieved in the “aged” stormwater and potable water cells. A worst-case scenario was assumed: no loss of heavy metals.

To achieve these target concentrations and minimise the introduction of artefacts through artificially contaminating the media [30], “naturally” contaminated sediment was introduced from a stormwater wetland in an industrial catchment (Newport, Melbourne, Australia) to age these gardens to their five- and ten- year metal equivalents. Because this sediment had a unique metal-concentration profile, it was not possible to achieve target concentrations for all the metals of interest. The mass of sediment added to each aged treatment was therefore optimised based on its dry weight, to set at zero the average percent deviation from the target concentrations for two potentially toxic metals: lead (Pb) and chromium (Cr). These two were selected as having most potential for harm. Pb, a non-essential toxic metal known to cause a variety of health problems [57]–[60], has been listed as a contaminant by Food Standards Australia New Zealand (FSANZ) [61]. Cr is potentially toxic, depending on its oxidative state and the quantity ingested. While trivalent Cr is recognised as an essential micronutrient, excessive ingestion has detrimental acute and chronic health impacts [62]–[63]. Moreover, oxidation of Cr to the hexavalent form makes it a carcinogen - thought to be harmful even in small doses [64]–[65]. Though cadmium (Cd) is recognised as a toxic non-essential metal, it was not included in the optimisation; it occurred in very low concentrations (≤1.55 mg/kg), so sediment input would need to be unrealistically high.

The mass of stormwater sediment added to the mixture (based on dried weights) depended on the age of the treatment and the water source (Table 2); the maximum being less than 16% of the media for the stormwater ten years aged treatment. The amount of composted manure soil was therefore also varied to ensure that the total volume of medium in the top layer equated to 0.027 m^3^ for all treatments. Sediment was sieved to remove all large organic material (>9 mm) before adding it to the composted manure soil. The top layer of medium for all aged treatments was prepared by measuring the appropriate amount of sediment and thoroughly mixing this into the measured mass of composted manure soil.

**Table 2:** The mass of sediment added in each cell, and the corresponding percentage volume.

Age (yrs)	Water source	Dry mass sediment (kg)	% volume sediment
**0**	Potable water	None	None
	Synthetic stormwater	None	None
**5**	Potable water	0.0136	0.11
	Synthetic stormwater	0.9	7.6
**10**	Potable water	0.0269	0.23
	Synthetic stormwater	1.87	15.7

Total volume of media in each cell was 0.027 m^3^.

Additional physico-chemical parameters of this top-layer mixture were also analysed for each of the different soil ages (Table 3). This was to ensure that aside from metal concentrations, the properties of the five- and ten-year aged soils (both potable and stormwater) were not dramatically different from the zero-year age soils. Outcomes of the simulated ageing process are presented in Figure S1 in File S1.

**Table 3:** The median starting soil metal concentrations for each aged treatment (*n* = 3).

Parameter	0^1^ yrs	Pot^a^ 5 yrs	Pot^a^ 10 yrs	SW^b^ 5 yrs	SW^b^ 10 yrs
Cd	<0.2	<0.2	<0.2	<0.2	0.3 [0.2, 0.3]
Cr	15 [10,16]	9 [9,14]	11 [10,12]	13 [11,13]	15 [15,16]
Pb	8 [8,9]	8 [8,10]	9 [9,22]	25 [23,25]	60 [56,67]
Cu	14 [14,16]	16 [16,16]	16 [15,16]	16 [16,16]	25 [22,25]
Fe	8600 [5700, 8600]	5500 [5300, 9700]	5600 [5400, 7200]	6900 [6500, 7500]	8600 [8400, 8800]
Zn	68 [60,70]	71 [69, 98]	74 [71,76]	200 [180, 200]	470 [410, 490]
pH	7.3 [6.7, 7.6]	7 [7, 7.1]	7.2 [7.1, 7.3]	6.8 [6.6, 6.9]	6.8 [6.7, 7]
EC^c^ (µS/cm)	975 [610, 1120]	1070 [720, 1280]	580 [520, 930]	1990 [1480, 2970]	1980 [1970, 2040]
TN^d^ (mg/kg)	6200 [4800, 6300]	5800 [5700, 6100]	6000 [5900, 6000]	6000 [5700, 6100]	5700 [3400, 6500]
TP^e^ (mg/kg)	2000 [1900, 2500]	2200 [2200, 2400]	2300 [2000, 2500]	1700 [1700, 1900]	1900 [1700, 1900]
Bulk density (kg/m^3^)	719 [690, 745]	724 [715, 749]	735 [726, 742]	803 [757, 839]	824 [819, 831]

Concentrations are from soil samples taken on 7 February, 2013.

Values in the square brackets represent minimum and maximum.

0 yrs, 5 yrs and 10 yrs signify the respective age groups.

1 ^1^Concentrations for 0 yrs are applicable to the synthetic stormwater and potable water irrigated zero year treatments (i.e. the composted manure soil with no sediment addition used for the SW 0 yrs and Pot 0 yrs).

a Pot  =  potable.

b SW  =  synthetic stormwater.

c EC  =  electroconductivity.

d TN  =  total nitrogen.

e TP  =  total phosphorus.

### Untreated synthetic stormwater

Synthetic stormwater was produced for the experiment to ensure a consistent and controlled laboratory environment and to avoid the logistical constraints associated with collecting urban stormwater runoff. To simulate a worst-case scenario, the literature was used to quantify the 90^th^ percentile of microorganism and metal concentrations for untreated urban stormwater (Table 4).

**Table 4:** Target and measured median concentrations of metal (*n* = 9) and *E. coli* (*n* = 20) in synthetic stormwater, and measured median concentrations of metal (*n* = 5) and *E. coli* (*n* = 5) in potable water.

Parameter^a^	Concentration (mg/L)			
	Target stormwater concentration^c^	Median in measured synthetic SW	Chemical addition to synthetic SW	Median of measured potable water
Al^b^	1.864	0.63 [0.18, 2.2]	1.85	0.045 [0.037, 0.057]
Cd	0.0095	0.013 [0.0054, 0.015]	0.01	0.00002 [0.00002, 0.00003]
Cr	0.0606	0.055 [0.027, 0.0762]	0.061	0.00066 [0.0002, 0.00073]
Cu	0.193	0.074 [0.047, 0.116]	0	0.029 [0.015, 0.35]
Pb	0.43	0.33 [0.119, 0.41]	0.43	0.00065 [0.00039, 0.0014]
Fe	8	6.2 [1.67, 8.68]	7.95	0.076 [0.055, 0.084]
Ni	0.066	0.052 [0.02, 0.0594]	0.066	0.00057 [0.00028, 0.0012]
Mn	0.089	0.093 [0.062, 0.136]	0.088	0.0069 [0.0062, 0.0077]
Zn	0.585	0.9 [0.502, 1.2]	0.562	0.01 [0.0039, 0.019]
*E. coli* (MPN^d^/mL)	242	258 [54.78, 681]	—	<1

Note that some measured values are less than the chemical mass added, due to possible dilutions caused by using an approximate filling technique for the tank.

Values in square brackets are for the 5^th^ and 95^th^ percentiles.

a Metals were added in the following chemical forms: AlCl_3_; Cd (solution, 1000 mg/L); Cr(NO_3_)_3_; PbNO_3_; FeCl_3_; Ni(NO_3_)_2_; Mn(NO_3_)_2_; ZnCl_2_.

b ALCl_3_ was not added in the first three irrigation events due to problems with supply.

c Values based on literature [6], [33]–[52].

d MPN  =  Most Probable Number.

The method described in Hatt et al. [66] was used to prepare the synthetic stormwater with a fresh batch produced for each dosing event. It was prepared in a round 5000 L Colorbond water tank fitted with an internal agitator rotating at 200 rpm and driven by a 1.5 kW electric motor. First, dissolved sodium thiosulphate was added to dechlorinate 600 L of tap water, and mixed in using the internal agitator. Then 3 L of freshly collected raw sewage (<1 day old, sourced from the Pakenham Water Recycling Plant) was added to seed the microorganisms - contributing also to the required levels of heavy metals. *E. coli* concentrations averaged 258 MPN/mL, slightly above the target concentration of 242 MPN/mL. Additional seeding was needed to meet the target concentrations of Cd, Cr, Pb, Cu, Al, Fe, Mn and Ni (Table 4).

### Dosing regime

As previously described, a weekly irrigation volume of 2.6 L per cell of 0.09 m^2^ was determined. This estimate is slightly above the Australian average irrigation water volume used in agriculture [67]. Irrespective of water source type, all crops were initially irrigated with potable water for 14 days after planting to facilitate establishment. Following this, each cell was irrigated twice a week with 1.3 L of their respective water source. On each dosing day, either synthetic stormwater or potable water was delivered to each cell in increments; that is, the systems were watered in three rounds (in the first and second rounds each cell received 500 mL and in the third round received 300 mL). This was to ensure that all cells received similar water quality. Although the stormwater tank was mixed mechanically, differences in water quality could occur as the tank emptied; and potable water quality could also vary over time.

The water was delivered to each cell using a perforated plastic container placed above the crops to mimic overhead sprinkler irrigation. Each dosing session was approximately 2 hours, and dosing was carried out for 11 weeks - equating to 21 dosing events from 25 February to 13 May, 2013.

### Sampling and processing

Synthetic stormwater was sampled a total of nine times throughout the experiment. Water samples were collected in three consecutive irrigation events towards the beginning and end of the experiment (15–21 March and 26 April-3 May 2013, respectively) and collected every 2 or 3 weeks during the middle of the experiment (21 March-26 April, 2013). Samples were taken by dispensing synthetic stormwater incrementally into three clean 5 L plastic containers throughout the irrigation period. After mixing, water from these three containers was combined in equal portions to obtain a composite sample for the dosing event. Potable water was sampled a total of five times throughout the experiment using a similar sample collection method. However, only one 5 L plastic container was used, given the relatively uniform quality of potable water.

Soil samples taken at the beginning (7 February 2013) and end (21–22 May 2013) of the experiment were collected using a 30 cm stainless steel metal core rinsed with de-ionised water. Soil samples consisted of a composite of three cores randomly selected around the crop, obtained from a depth of 0–15 cm. This depth was selected because concentrations of metals are higher in surface soils [16], [20], [68]. The metal core was thoroughly rinsed with de-ionised water before each individual sample collection. Soil and water samples were refrigerated, and then transported to a laboratory accredited by the National Association of Testing Authorities (NATA) for analysis by inductively coupled plasma mass spectrometry (ICP-MS) using standard procedures specified by the US Environmental Protection Agency [69].

Crops were harvested, along with their corresponding soil samples, after 13 weeks of the experiment. At least 150 g of the edible part of each crop was sampled - including kale leaves, French bean pods, beetroot leaves (called beetleaf, below) and beetroot bulbs (called simply beetroot, below). Gloves were worn during the collection of the samples. Each sample was rinsed with 800 mL of de-ionised water to remove any superficial contaminants -from atmospheric deposition for example [16]–[17], [70] - and then placed in an individual plastic ziplock bag. The samples were then refrigerated and transported for analysis at the National Measurement Institute (NMI, Melbourne, Australia). They were processed using standard analytical procedures [69], [71] by ICP-MS (Agilent 7500CE).

### Data analysis

Bioconcentration factors, measuring the ability of plants to uptake and transport metals to their biomass, were computed for all crops [72]–[73]. To meet the objectives of this study, the *bioconcentration factor* was calculated based on the metal concentrations in the edible components-:
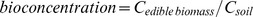
where *C_edible biomiass_* is the concentration found in the edible biomass (mg/kg) and *C_soil_* is the concentration found in the soil (mg/kg). Crop metal concentration and bioconcentration data were analysed using statistical analysis software (SPSS, USA). These variables were log-transformed before analysis, because of their log normal distributions.

Statistical significance and significant interactions were identified using factorial analysis of variance (ANOVA). The level of significance was set at *P≤*0.05. The mean, median and range of metal concentrations in the crops and soil were also computed, along with the associated bioconcentration factors.

## Results and Discussion

### Uptake of metals in various crops

**Figure 1:**
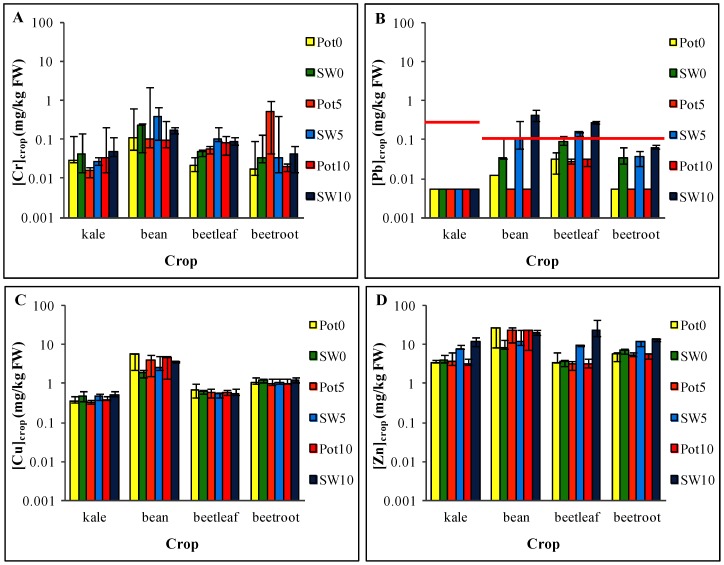
Median metal concentrations in edible biomass of all potable and stormwater irrigated crops. Concentrations for (A) Cr, (B) Pb, (C) Cu and (D) Zn. The legislative maximum levels [57] of Pb are indicated by the red line. The bars represent maximum and minimum concentrations recorded. *Note:* FW  =  fresh weight; Pot  =  potable water; SW  =  stormwater; the numbers 0, 5 and 10 in the legend represent the age groups 0 years, 5 years and 10 years, respectively; samples below the detection limit (<0.01 mg/kg) are presented at a concentration of 0.005 mg/kg as per World Health Organisation (1995) http://www.who.int/foodsafety/publications/chem/en/lowlevel_may1995.pdf.

The concentration of heavy metals in the edible portion of each crop varied significantly according to crop type (Figure 1). In most cases, French bean differed significantly from all other crops (e.g. Tukey's post hoc *p*≤0.05 for Cu) while concentration in kale and beetleaf were the most similar. Zn had the highest concentrations for most crops, ranging from 2.2 mg/kg (beetleaf; potable water, aged five years) to 43 mg/kg (beetleaf; synthetic stormwater, aged ten years); and Cd had the lowest concentrations, ranging from <0.01 mg/kg to 0.043 mg/kg. The variability between crop types (Figure 1), and also across metal concentrations within each crop type, appears to be typical of metal uptake by plants [16], [29], [74]–[77]. This variability is often strongly linked to specific plant characteristics, and to concentrations of bioavailable heavy metals in the soil.

**Figure 2:**
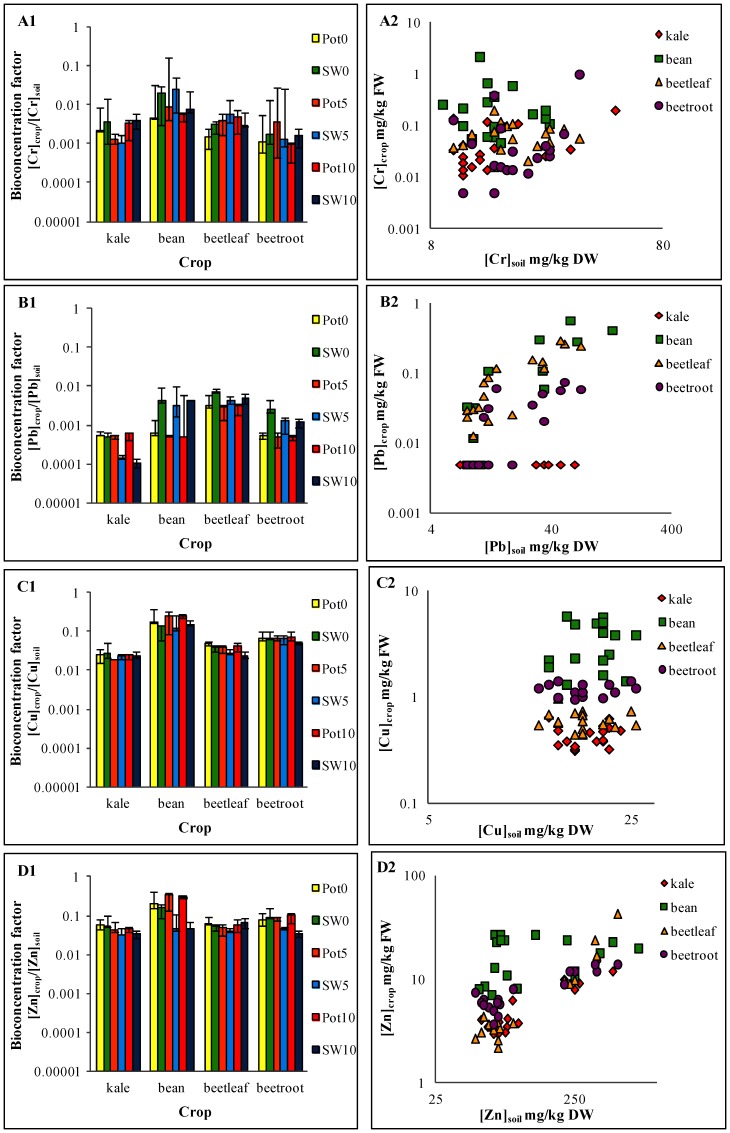
Bioconcentration factor for all crops (A1–D1) and relationship between soil and crop metal concentrations (A2–D2). Bioconcentration factors, soil and crop concentrations are for (A) Cr, (B) Pb, (C) Cu and (D) Zn. Bioconcentration factors are the median for all potable and stormwater irrigated crops. The bars represent maximum and minimum concentrations recorded (*n = *3). *Note:* Pot  =  potable water; SW  =  stormwater; the numbers 0, 5 and 10 in the legend represent the age groups 0 years, 5 years and 10 years, respectively; bioconcentration factor for crop samples below the detection limit (<0.01 mg/kg) was calculated as half the detection limit (0.005 mg/kg) divided by the soil concentration as per World Health Organisation (1995) http://www.who.int/foodsafety/publications/chem/en/lowlevel_may1995.pdf.

To further explore the differences in crop types, it is useful to compare the concentration of heavy metals in each crop to the concentrations in the soils. Figure 2 presents both bioconcentration factors (Figure 2 A1–D1) and scatter plots of crop versus soil concentrations (Figure 2 A2–D2), emphasising the degree of variability exhibited across crop type and also across metals within a crop type. Indeed, Figure 2 A1–D1 shows that bioconcentration factors for French bean range from 0.34 (Zn; potable, aged five years) to 0.0005 (Pb; potable, aged ten years); and for beetroot from 0.001 (Cr; potable, aged ten years) to 0.1 (Zn; potable, aged ten years). The uptake rates for each crop type within this experiment (in descending order) were as follows: French bean> beetleaf> beetroot> kale. Although this order is generally consistent with the literature, our legume (French bean) crop's metal uptake behaviour is not typical of what is reported in previous studies [29], [76], [78]–[80]. Figure 2 A2–D2 illustrates that uptake of Pb and Zn in most crops increases slightly with an increase to soil concentration, however this increase is non-linear. In contrast, the uptake patterns of Cr and Cu do not show this positive response to increasing soil concentration.

French bean exhibits the highest bioconcentration rates, as inferred from its high observed metal concentrations, and generally demonstrates the greatest deviation from the other crop types (e.g. Tukey's post hoc *p*≤0.05 for Cr). Many studies have classed leguminous species as low metal accumulators [29], [80]–[81]. However, French bean (*Phaseolus vulgaris*) has been identified as a species that can accumulate large amounts of Pb compared to other leguminous species. It has also been suggested for use in the phytoextraction of Pb from polluted waters [82]. It is possible that this increased uptake may be facilitated by symbiotic rhizobia that are able to release metabolites to increase plant access to metals [83]–[85]. Indeed, in addition to metal bioavailability, plants are known to be able to regulate or promote metal uptake through the secretion of sequestering agents [86]–[88].

In contrast, the result of our experiment demonstrate that kale held very low concentrations and bioconcentration factors (Figures 1 and 2), and was, surprisingly, one of the lowest Pb accumulators. Despite the reputation for species in the *Brassicacea* family to be metal hyper-accumulators, many studies have reported considerable variation in the uptake behaviours within this family. For example, from their investigation of metal contamination in crops grown near a smelter, Cui et al. [16] recommended the cultivation of choy sum (*Brassica campestris* var. *parachinensis*) in Pb-contaminated soils because of its low rate of Pb accumulation. Conversely, they discouraged the use of leaf mustard (*Brassica juncea*) because of its Pb-accumulating capabilities. Fang et al. [75] identified Chinese kale (*Brassica alboglabra*) as a species that was non-reactive (thus metal-resistant) to high uptake of Pb and Cu from their studies on uptake in metal-contaminated phosphorus-amended soils. The uptake behaviour of kale in our experiment suggests that it may be actively or incidentally restricting the movement of Pb and other metals. This behaviour was also observed in rape (*Brassica napus*) by Angelova et al. [23], who found limited transport of Pb to aerial biomass. They also generally found Cd readily translocating from roots to shoot and accumulating in the above-ground biomass; but while limits on the detection of Cd in the soils preclude a strong conclusion, kale yielded no detectable Cd (in contrast to our other crops). This suggests that kale is similarly restricting the movement of Cd.

These distinct crop metal uptake behaviour are reflected in the significant interaction between water source and crop type for Pb, Cu and Zn when using bioconcentration (*p*≤0.05). An increase in the available dissolved metals occurs when the water source changes from potable to stormwater. But then, some crops actively take up newly available metal cations (such as French bean) and some restrict any uptake (such as kale) - reinforcing the conclusion that different plants have different uptake mechanisms.

### Meeting Australian food standards

In Australia, food quality is regulated under the Food Standards of Australia and New Zealand (FSANZ) [61]. For metal contamination pertaining to vegetables, these standards set a maximum permissible limit of 0.1 mg/kg for Cd and Pb that applies to all vegetables except *Brassicas*, which are permitted 0.3 mg/kg for Pb. Comparison of the crop metal concentrations in this study against these standards highlights that a number of our crops exceeded the permissible limit for Pb. This experiment rendered six of nine French bean samples and seven of nine beetleaf samples over this limit with concentrations ranging from 0.11 to 0.58 mg/kg (French bean) and 0.12 to 0.3 mg/kg (beetleaf). Currently, the maximum exposure limits for Pb are being reviewed [89].

Interestingly, all kale samples were below the detection limit for Pb (<0.01 mg/kg). While Pb accumulation increases with soil age, the FSANZ [58] maximum permissible limit for Pb is breached even in the zero year soils planted with French bean and beetleaf. These results suggest that consumption of French bean pods beetleaf in conjunction with other forms of exposure to Pb (such as consumption of other foods contaminated with lead, exposure to aerosols, or smoking), may constitute a health risk.

### Influence of water source type

To identify the effects of water source without the confounding influence of system age, we analysed data collected from the zero-year treatments. Water source was only a significant factor for crop concentration and bioconcentrations for Pb (*p*≤0.05) with concentrations significantly higher in crops irrigated with stormwater than those irrigated with potable water (Figure 1 B). Therefore, a larger pool of bioavailable metals is available for crops irrigated with stormwater compared to the equivalent crops irrigated with potable water. This is reflected in the higher Pb-bioconcentration values for stormwater-irrigated crops compared to potable water-irrigated crops (Figure 2 B2). The ratio of the total Pb mass applied via irrigation to the final Pb concentration in the soil is 0.8 for stormwater, compared to 0.0021 for potable water. The same calculation for all other metals yields a smaller difference between the two water types. Stormwater and potable water ratios for Cu were 0.13 and 0.086; and for Zn, 0.33 and 0.0038. As our synthetic-stormwater metal concentrations were based on measured values reported in the literature (Table 4), these findings highlight where the impact of stormwater is likely to be observed. They suggest that they influence of irrigation with stormwater is not apparent in metals where the concentration difference between stormwater and potable water may be minimal *and* the metal is naturally abundant in the media, thereby dwarfing any tangible changes stemming from stormwater irrigation.

The influence of water source was not significant for Cr crop metal concentration, or for bioconcentration. This is likely to be a result of the variability in the Cr soil concentrations for both potable and stormwater (see File S1). There are instances where Cr soil concentrations are higher for potable water compared to stormwater and vice versa, without any clear patterns emerging to distinguish water sources or age groups. This variability has translated into crop concentrations (Figure 1 A) and consequently the Cr bioconcentration factors (Figure 2 A1).

### Influence of vegetable garden age

The influence of vegetable garden age was significantly only for the crops irrigated with stormwater (Pb and Zn, *p≤*0.05), both in terms of crop metal concentration and bioconcentration. The absence of such a relationship between age and the crops irrigated with potable water may reflect the marginal differences in the soil metal concentrations between age groups (see File S1). For stormwater treatments, there is an observable decrease in bioconcentration with increasing vegetable garden age (Figure 2 A1–D1), rendering it significant for Pb, Zn (*p*≤0.05) and almost significant for Cu.

The decrease in bioconcentration with increasing age (Pb and Zn, Figure 2 A1 and D1) highlights the non-linear nature of plant uptake of metals (Figure 2 A2–D2). Studies have found that while crop metal concentrations are generally indicative of ambient soil concentrations [17], [20], [90]–[91], the concentration gradients in the soil and in the plant are very different [16]–[17], [19]–[20], [73]. This is attributable to two factors: plant uptake rates and bioavailable metal mass. Our results demonstrate that plant uptake gradients vary across metals (Figure 2 A2–D2). For some metals such as Cr and Cu (Figure 2 A2 and C2), uptake rates remain fairly stable despite the marked increases in the soil concentration with increasing age. Conversely, for Pb and Zn (Figure 2 B2 and D2), increases to concentration in the edible biomass tend to align with increase in the soil metal concentration, although the gradients differ. This may be in part due to the plants themselves regulating their own uptake of metals [23], and also the bioavailable metal mass. As metal residence time in soil increases, it facilitates binding with different soil fractions through time-dependent diffusion, absorption, hydrolysis and precipitation processes [26]–[28]. This reduces availability of metals and changes their ecotoxicity potential [29]–[30].

Studies using artificial spiking to generate contaminated soils for ecotoxicity assessments have acknowledged the tendency to overestimate risks due to the larger pool of bioavailable metal cations [29]–[30]. In preference to ageing the cells by artificial spiking, our experiment used previously contaminated stormwater sediment collected from an irregularly maintained stormwater wetland that has treated industrial runoff for decades. Due to the increased residence time of these sediments, it is likely that the pool of bioavailable metals in these soils is less than in comparable artificially spiked soils. Despite increases in the total soil metal concentration with garden age, the bioavailable pool may not, therefore, have increased at the same rate. The addition of the contaminated sediment to the aged treatments may have also contributed to this phenomenon. The sediment has increased the bulk density in the ten year aged treatments by approximately 15% when compared to the zero year equivalents (see Table 3). This artefact may have created an artificial restriction in root growth and distribution, limiting its potential to uptake bioavailable metals in comparison to crops growing in the zero year aged treatments.

### Comparison to bioconcentration factors in the literature

Bioconcentration factors determined in this study were compared with those from previous studies that have investigated food crops irrigated by alternative water sources (Table 5). Our study found bioconcentration ranges resembling those of the only other study that focussed on stormwater [31]; but studies of other water sources found much higher ranges. These differences may be linked to localised ambient air and soil pollution levels [94], and may also be influenced by variations in crop species or physico-chemical qualities of the media. Nonetheless, the considerably lower bioconcentrations factors observed in our study and those of McCarthy et al. [31] emphasise the importance of water quality characteristics on the uptake of metals into food crops.

**Table 5:** Comparison of the bioconcentration factor range in the literature for crops irrigated with various contaminated water sources.

Source	Water type	Crop type	Bioconcentration range (min to max)			
			Cr	Pb	Cu	Zn
This study^a^	urban stormwater	root, leafy, *Brassica* and leguminous	0.00078–0.049	<0.00008–0.01	0.02–0.25	0.026–0.197
McCarthy et al. 2011 [29]	urban stormwater	root and leafy veg	<0.0019–0.02	<0.001–0.11	0.016–0.66	0.038–0.145
Khan et al. 2008^b^ [16]	biologically treated wastewater	grain, leafy, *Brassica* and root	0.08–0.38	0.02–0.13	0.16–0.85	0.16–0.53
Rattan et al. 2005^b3^ [19]	effluent from sewage treatment plant	grain, leafy, *Brassica*, root, fruiting	not tested	not tested	1.69–12.9	7.25–24.6
Rattan et al. 2005^b3^ [19]	contaminated ground water	grain, leafy, *Brassica*	not tested	not tested	2.48–18.1	21.2–55.6
Khan et al. 2013^b^ [92]	contaminated stream water^1^	grain, herbaceous, bulbous, fruiting, root	0.01–0.22	0.52–1.50	not tested	not tested
Khan et al. 2013^b^ [92]	contaminated ground water	grain, herbaceous, bulbous, fruiting, root	<0.00^2^–0.18	0.91–3.96	not tested	not tested
Liu et al. 2005^b3^ [93]	river water receiving wastewater effluent	grain, herbaceous, *Brassica*, leafy	0.01–0.19	0.12–0.23	0.15–0.86	0.42–0.95

# ^a^Reported values represent only crops irrigated by synthetic stormwater from all age groups (zero-, five- and ten-year aged treatments).

b Analysis based on dry weight of crops.

1 Contaminated with untreated industrial effluent, urban and domestic sewage and municipal waste.

2 Limit of significant figures provided.

3 Range of maximum and minimum of means for each crop species analysed.

### Outcomes for practice

We deliberately set out to test a worst-case scenario, using untreated as opposed to treated urban stormwater. We found that Pb accumulation can occur in some crops, such as French bean and beetleaf, to the point that concentration exceeds permissible limits set by Australian and New Zealand standards [61]. Our results suggest that consumption of French bean or beetleaf in conjunction with other forms of exposure to Pb (such as smoking, other foods contaminated with Pb, and aerosols) may constitute a health risk. It is therefore important that assessments of risk be holistic: they should include all forms of Pb exposure, to understand risk from consumption of produce irrigated with urban stormwater in the context of risk from all sources combined (e.g. refer to Figure S2 in File S1 for an approximate calculation of Daily intake of metals).

Our results also show, however, that crop selection plays an important role in metal uptake, and that this is critical for restricting accumulation of Pb specifically. It may therefore be feasible to use untreated stormwater if careful research on the metal uptake properties of crop species (and particular cultivars) is undertaken. Metal-resistant species such as kale or others that accumulate less metal, such as root or fruiting crops [76], [78], [95] - may be safer to grow when stormwater is used.

Attention should also be given to the root interactions between crops that are co-cultivated in such circumstances, as this may increase metal contamination in normally low-uptake crops. For example, Liu et al. [96] found that in most cases, several species of legumes (Japanese clover, cowpea and soybean) co-cultivated with other crops (tomato, maize, pak choi and cabbage) resulted in increased Cd concentrations in the edible biomass of these crops when compared to monocultures of the same crops. This phenomenon was attributed partly to these legumes' local reduction of soil pH, allowing for a higher exchangeable Cd fraction. Such results highlight the importance of careful selection and co-cultivation of crops in determining the extent of metal contamination, suggesting a particular line of future research with crops irrigated with stormwater (with a focus on Pb accumulation).

In circumstances where untreated stormwater is to be used, existing media should be “diluted” with new, non-contaminated media to reduce metal concentrations, and to maintain metal accumulation at a minimum. However, to eliminate the possibility of Pb accumulation and to enable full flexibility in terms of crop selection, it is recommended that urban stormwater be treated before use in irrigation.

## Conclusion

Our study investigated metal contamination of kale, French bean and beetroot crops when they are irrigated with untreated urban stormwater. We have found that Pb is the most problematic metal, and that its accumulation in crops can exceed Australian standards. Metal accumulation in crops increases with increase in irrigation longevity, though not at the same rate. Our findings also highlight that kale is capable of restricting metal uptake or translocation (or both) to edible biomass, making it safe to consume despite the use of a contaminated water source. This study is the first of its kind to demonstrate the implications of using stormwater as an irrigation source, and to finding that it is indeed feasible if an understanding of the risks is used to implement appropriate safeguards.

The real-world implications for public health and for safe cost-effective use of a readily available water resource are considerable. We would advocate strongly for continued research along the lines laid down in the present study - perhaps with a wider range of food crops and treatments - to develop even more secure recommendations for practice.

## Supporting Information

File S1 **Supplementary results and discussion for baseline soil metal concentrations and daily intake of metal, also containing Figure S1 and Figure S2.** Figure S1, Median soil metal concentrations from soil samples taken at the beginning (7^th^ February, 2013) and end of the experiment (21^st^–22^nd^ May, 2013) (*n = *9). Note that the median for the end samples is from a larger sample size combining all crops. The bars represent maximum and minimum concentrations recorded. *Note:* BL  =  baseline (ie. start of experiment); Pot  =  potable; Sw  =  semi-natural stormwater; the numbers 0, 5 and 10 in the legend represent the age groups 0 years, 5 years and 10 years, respectively. Figure S2, Daily intake of metals for the average Australian adult and the corresponding Provisional Maximum Tolerable Daily Intake levels (PMTDI). Notes: the PMTDI is denoted by the red line; there is currently no upper intake limit for lead with the dashed red line representing the previous PMTDI of 0.0036 mg/kg of body weight per day (now withdrawn). *Sources:* Expert Group on Vitamins and Minerals (2003) Safe upper levels for vitamins and minerals. Available: http://cot.food.gov.uk/pdfs/vitmin2003.pdf, Accessed 13 September, 2013; World Health Organisation (2010) Evaluations of the Joint FAO/WHO Expert Committee on Food Additives (JECFA). Available: http://apps.who.int/food-additives-contaminants-jecfa-database/search.aspx, Accessed 13 September, 2014.
(DOCX)
