# Supplementary material for: Heavy Metal Contamination of Vegetables Irrigated by Urban Stormwater: A Matter of Time?
Source: PLoS One. 2014 Nov 26;9(11):e112441. doi: 10.1371/journal.pone.0112441 (PMC4245087; doi:10.1371/journal.pone.0112441)
Supplement: File S1 — Supplementary results and discussion for baseline soil metal concentrations and daily intake of metal, also containing Figure S1 and Figure S2. Figure S1, Median soil metal concentrations from soil samples taken at the beginning (7th February, 2013) and end of the experiment (21st–22nd May, 2013) (n = 9). Note that the median for the end samples is from a larger sample size combining all crops. The bars represent maximum and minimum concentrations recorded. Note: BL = baseline (ie. start of experiment); Pot = potable; Sw = semi-natural stormwater; the numbers 0, 5 and 10 in the legend represent the age groups 0 years, 5 years and 10 years, respectively. Figure S2, Daily intake of metals for the average Australian adult and the corresponding Provisional Maximum Tolerable Daily Intake levels (PMTDI). Notes: the PMTDI is denoted by the red line; there is currently no upper intake limit for lead with the dashed red line representing the previous PMTDI of 0.0036 mg/kg of body weight per day (now withdrawn). Sources: Expert Group on Vitamins and Minerals (2003) Safe upper levels for vitamins and minerals. Available: http://cot.food.gov.uk/pdfs/vitmin2003.pdf, Accessed 13 September, 2013; World Health Organisation (2010) Evaluations of the Joint FAO/WHO Expert Committee on Food Additives (JECFA). Available: http://apps.who.int/food-additives-contaminants-jecfa-database/search.aspx, Accessed 13 September, 2014. (DOCX) [file pone.0112441.s001.docx]

**File S1**

**Soil metal concentrations**


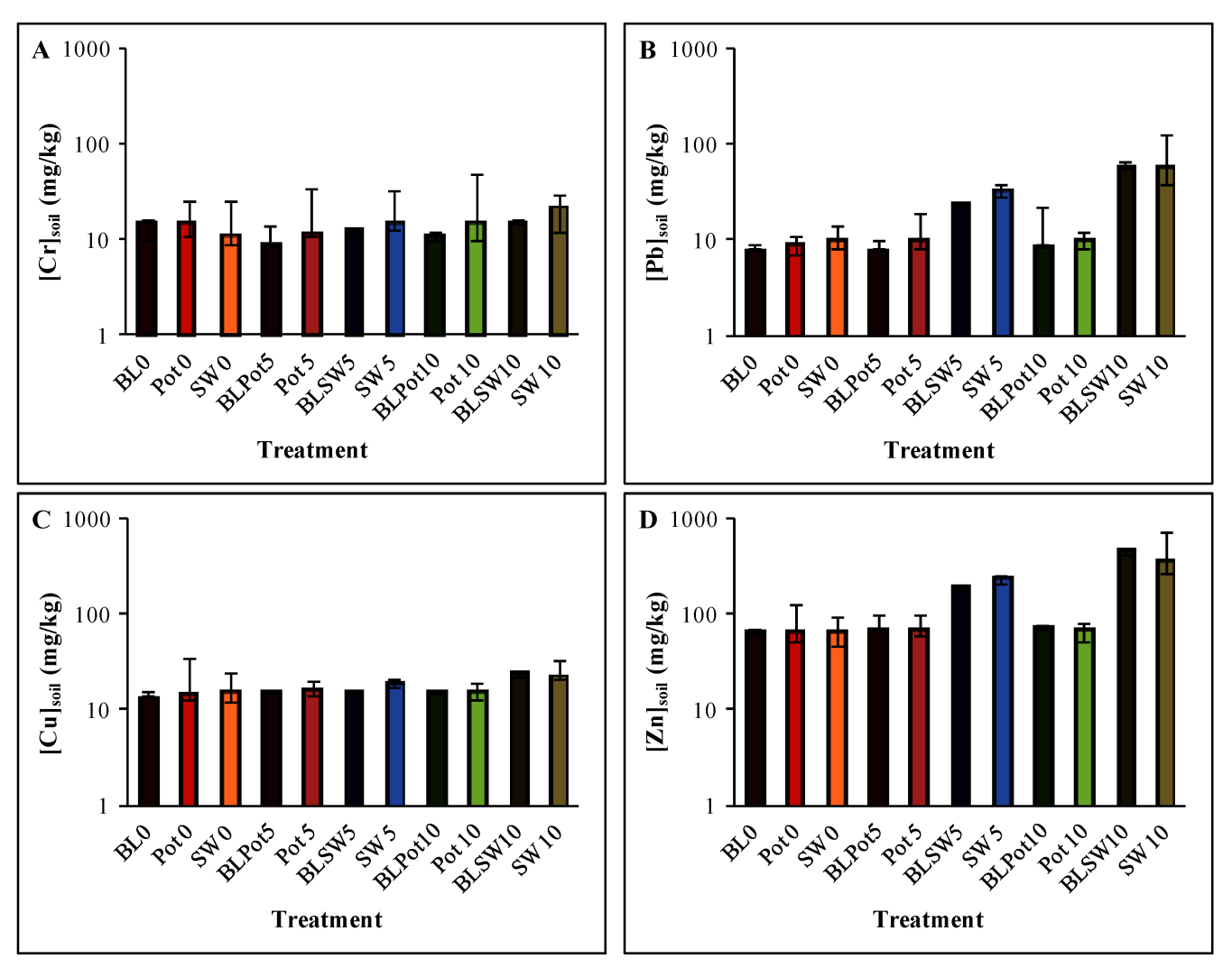


**Figure S1.** **Median soil metal concentrations from soil samples taken at the beginning (7^th^ February, 2013) and end of the experiment (21^st^ – 22^nd^ May, 2013) (*n =* 9).**

Note that the median for the end samples is from a larger sample size combining all crops. The bars represent maximum and minimum concentrations recorded. *Note:* BL = baseline (ie. start of experiment); Pot = potable; Sw = semi-natural stormwater; the numbers 0, 5 and 10 in the legend represent the age groups 0 years, 5 years and 10 years, respectively.

Baseline soil metal concentrations measured at the beginning of the experiment did not meet the target metal concentrations (Figure S1). This difference was a result of the fixed concentration profile of sediment added to aged media. Deviation from target concentrations was highest for the stormwater aged treatments, ranging from a 21% underestimate (Cr) to 290% overestimate (Zn). However, it is important to note that although there was deviation from the target concentrations, the starting concentrations nevertheless mimicked patterns indicative of the ageing process for stormwater-irrigated vegetable gardens. These patterns included:

- increasing metal concentration with increasing age
- establishing only marginal differences in metal concentrations between the potable water irrigated aged treatments
- establishing substantial differences in metal concentration between the stormwater irrigated aged treatments.

Thus the ageing method used was able to mimic the natural metal accumulation patterns expected from long-term stormwater and potable water irrigation within the context of realistic risk boundaries.

Figure S1 compares the final soil metal concentrations with the starting concentrations, and highlights that, in most cases, the former is at least marginally higher than the baseline concentrations. The absence of this trend for Cr and Cu is most likely associated with substantial differences between concentrations in the synthetic stormwater and soil, rather than being a product of the experiment’s design. Ambient concentrations of Cr and Cu in the manure compost are relatively high compared to the two water sources, which means that irrigation with these water sources, particularly synthetic stormwater, would not lend itself to measureable changes in the soil concentrations. This trend reflects reality, whereby metals that are naturally abundant in garden soils, and occur in lower concentrations in irrigation water, will show little if any increases in concentration.

**Daily intake of metal for each stormwater irrigated aged treatment**

To identify the health implications, the daily intake of metal and the hazard quotient were computed using the following equation [15]:

*Daily intake of metal (DIM)* = [metal](mg/kg) × conversion factor × average daily intake of veg (kg/person/day)/body weight (kg)

where conversion factors of 0.129, 0.1603, 0.194 and 0.11 were used for kale, French bean, beetroot and beetleaf respectively. The average daily intake was taken to be 0.375 kg, following the Recommended Dietary Intake (RDI) levels set by the National Health and Medical Research Council [1]. A DIM was calculated for each crop separately; for simplicity, it was assumed that the total RDI of vegetables was achieved through consumption of each of the crops. An average body weight of 78.5kg was assigned to represent an average Australian adult [2].

Figure S2 shows the results of the DIM levels and compares them to the Provision Tolerable Maximum Daily Intake (PTMDI) levels set by WHO. It shows that in all cases, the consumption of these crops is far below the PTMDIs. However, the results for Pb need to be treated with caution, as the current PMTDI has been withdrawn, and a new threshold has not yet been established.


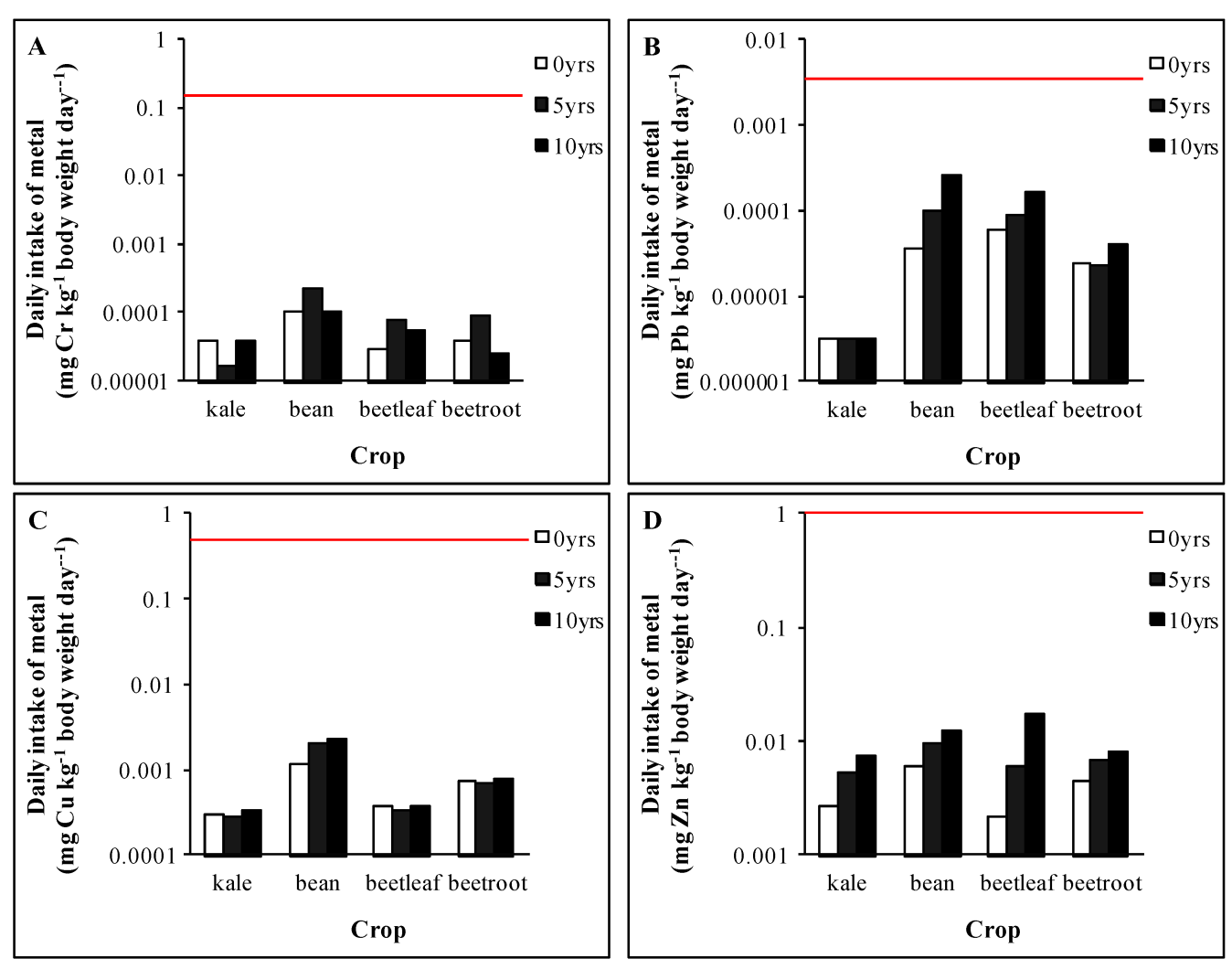


**Figure S2.** **Daily intake of metals for the average Australian adult and the corresponding Provisional Maximum Tolerable Daily Intake levels (PMTDI).**

Notes: the PMTDI is denoted by the red line; there is currently no upper intake limit for lead with the dashed red line representing the previous PMTDI of 0.0036mg/kg of body weight per day (now withdrawn). *Sources:* Expert Group on Vitamins and Minerals (2003) Safe upper levels for vitamins and minerals. Available: <http://cot.food.gov.uk/pdfs/vitmin2003.pdf>, Accessed 13 September, 2013; World Health Organisation (2010) Evaluations of the Joint FAO/WHO Expert Committee on Food Additives (JECFA). Available: <http://apps.who.int/food-additives-contaminants-jecfa-database/search.aspx>, Accessed 13 September, 2014.

**Sources**

1. National Health and Medical Research Council (NHMRC) (2013) Eat for health: Australian dietary guidelines. Australian Government. Available: <http://www.nhmrc.gov.au/_files_nhmrc/publications/attachments/n55_australian_dietary_guidelines_0.pdf>, Accessed 14 September, 2013.
2. Australian Bureau of Statistics (ABS) (2012) 4364.0.55.001 Australian health survey: first results, 2011‒12. Australian Government. Available: <http://www.abs.gov.au/ausstats/abs@.nsf/Lookup/4364.0.55.001main+features12011-12>, Accessed 14 September, 2013.
